# Hemoglobin levels are associated with retinal vascular caliber in a middle-aged birth cohort

**DOI:** 10.1038/s41598-024-59688-y

**Published:** 2024-04-20

**Authors:** Samuli Sakko, Mikko Karpale, Joona Tapio, Iina Leppänen, Oona Ahokas, Ville Saarela, M. Johanna Liinamaa, Peppi Koivunen

**Affiliations:** 1 Biocenter Oulu and Faculty of Biochemistry and Molecular Medicine, Oulu Center for Cell-Matrix Research, University of Oulu, P.O. Box 5400, 90014 Oulu, Finland; 2 Department of Ophthalmology, Medical Research Center Oulu and Research Unit of Clinical Medicine, Oulu University Hospital, University of Oulu, Oulu, Finland

**Keywords:** Medical research, Risk factors

## Abstract

Vascular and neural structures of the retina can be visualized non-invasively and used to predict ocular and systemic pathologies. We set out to evaluate the association of hemoglobin (Hb) levels within the national reference interval with retinal vascular caliber, optical coherence tomography (OCT) and visual field (VF) parameters in the Northern Finland 1966 Birth Cohort (n = 2319, 42.1% male, average age 47 years). The studied parameters were evaluated in Hb quintiles and multivariable linear regression models. The lowest Hb quintile of both sexes presented the narrowest central retinal vein equivalent (CRVE) and the healthiest cardiometabolic profile compared to the other Hb quintiles. In the regression models, CRVE associated positively with Hb levels in both sexes, (*B*_males_ = 0.068 [0.001; 0.135], *B*_females_ = 0.087 [0.033; 0.140]), after being adjusted for key cardiometabolic and inflammatory parameters, smoking status, and fellow vessel caliber. No statistically significant associations of Hb levels with central retinal artery equivalent, OCT or VF parameters were detected. In conclusion, Hb levels were positively and specifically associated with CRVE, indicating that Hb levels are an independent factor affecting CRVE and the effect is in parallel with established risk factors for cardiometabolic diseases.

## Introduction

Retinal microcirculation can be easily and non-invasively studied by fundus photography^1^. Alterations of retinal vascular diameter (RVD) but also of retinal nerve fiber layer (RNFL) thickness or macular parameters—likewise viewed non-invasively in vivo—may indicate prevalent ocular and systemic diseases such as hypertensive retinopathy and cardiovascular diseases (CVDs)^2,3^. Therefore, these parameters may have diagnostic or predictive value in managing diseases^4–6^.

The two components of RVD, arterial caliber, assessed as central retinal artery equivalent (CRAE), and venular caliber, assessed as central retinal vein equivalent (CRVE), associate both independently and together with adverse metabolism and metabolic diseases^3^. Narrower CRAE associates with hypertension whereas wider CRVE associates with hyperglycemia, obesity, and inflammation^3^.

We have previously shown that high hemoglobin (Hb) levels within the reference interval associate with adverse body composition and metabolic dysfunction^7^. High Hb levels also predict poorer cardiovascular health, increased total mortality and higher prevalence of gestational diabetes^7–9^. Hb levels vary by age, sex, race, living altitude, and smoking but are relatively stable at an individual level throughout adult life and therefore offer an intriguing window to personal metabolic disease risk^10^.

In elderly adults, CRAE and CRVE have been shown to associate with Hb levels^11–13^. As Hb levels alone associate with an adverse metabolic profile and poor cardiovascular health, we set out to study the association of Hb levels with vascular and neural parameters of the retina in a middle-aged Finnish birth cohort, and to evaluate whether this association is independent of cardiometabolic factors and fellow vessel caliber.

## Materials and methods

### Study population

A flow chart of the study population is presented in Fig. S1 of the supplementary materials. The study population is a subpopulation of the Northern Finland Birth Cohort 1966 Eye Study (NFBC1966 Eye Study, Fig S1), which is a randomized prospective cohort study conducted on a subpopulation of the original Northern Finland 1966 Birth Cohort (NFBC1966)^14,15^. The original NFBC1966 cohort consists of 12,058 subjects born live in the two northernmost provinces of Finland with an expected birth date in 1966 (Fig. S1)^15^. All subjects in the birth cohort aged 46 and 48 years old with a known postal address in Finland were invited to extensive clinical examinations including ophthalmological examinations. In the NFBC Eye Study, the subjects were randomized to a screening group (n = 5155) and a control group (n = 5166) (Fig. S1)^14^. Of the screening group, a total of 3,070 participants (60%) took part in the ophthalmological examination (Fig. S1)^14^. The study population for the current study (n = 2319, 42.1% males) consisted of the subjects of the screening group with ophthalmological measurements, smoking status and blood pressure (BP) data available and who had Hb levels within the Finnish reference interval (117–155 g/L for women and 134–167 g/L for men)^16^ (Fig. S1).

The study was conducted in accordance with the declaration of Helsinki and approved by the ethical committee of Northern Ostrobothnia Hospital District in Oulu, Finland. Written informed consent was provided by all participants.

#### Lifestyle and clinical parameters

Participants self-reported their smoking habits and smoking was classed as “non-smokers”, “past smokers”, or “current smokers”. Non-smokers were used as the reference group. Body weight and height were measured twice using regularly calibrated digital scales and stadiometers and the mean was used in the analyses. Body mass index (BMI) was calculated as the ratio of weight and height squared. Brachial systolic BP (SBP) and diastolic BP (DBP) were measured in the right arm of sitting participant three times with 1 min intervals after 15 min of rest using an automated oscillometric BP device and appropriately sized cuff (Omron Digital Automatic BP Monitor Model M10-IT). The average of the two lowest SBP values and corresponding DBP values were used in the analyses.

#### Laboratory measures

Blood samples were taken after an overnight fasting period, centrifuged and analysed immediately in NordLab Oulu (former name Oulu University Hospital, Laboratory), a testing laboratory (T113) accredited by Finnish Accreditation Service (FINAS) (EN ISO 15,189). Complete blood cell count including blood Hb level was determined from fresh samples using automatic electronic cell counter (Coulter Corp., Miami, FL) using spectrophotometric methods; red blood cells being measured using electric resistance detecting methods (impedance technology) with hydrodynamic focusing. Fasting plasma glucose and serum insulin levels were analysed by an enzymatic dehydrogenase method (Advia 1800, Siemens Healthcare Diagnostics, Tarrytown, NY, USA) and by a chemiluminometric immunoassay (Advia Centaur XP, Siemens Healthcare Diagnostics, Tarrytown, NY, USA), respectively. To evaluate insulin resistance, we calculated the homeostatic model assessment for insulin resistance (HOMA-IR) (fasting plasma glucose x fasting serum insulin/22.5). A two-hour oral glucose tolerance test (OGTT) was performed after an overnight (12 h) fasting period. Glucose and insulin values from OGTT were used to calculate Matsuda index for insulin sensitivity (10 000 × ((fasting plasma glucose × fasting serum insulin) × (fasting plasma glucose + 30 min plasma glucose + 60 min plasma glucose + 120 min plasma glucose)/4) × ((fasting serum insulin + 30 min serum insulin + 60 min serum insulin + 120 min serum insulin)/4))). Serum cholesterol (total, HDL, and LDL) and triglycerides were determined using an enzymatic assay method (Advia 1800; Siemens Healthcare Diagnostics Inc., Tarrytown, NY, USA). High sensitivity C-reactive protein (HsCRP) was analysed by an immunonefelometric assay (BN ProSpec, Siemens Healthcare Diagnostics Inc., Newark, DE, USA). Serum albumin, urea and creatinine concentrations were measured by Roche/Hitachi Modular Analyzer. Estimated glomerular filtration rate (eGFR) was calculated with CKD-EPI formula exploiting serum creatinine values^17^.

#### Ophthalmological measurements

Refractive error was measured for both eyes with an automated refractometer (Nidek AR-360A; Eye & Health Care Nidek Co., Ltd., Aichi, Japan). Fundus photos, OCT images and visual fields were obtained from both eyes for all the subjects. The left eye was used only if the quality of fundus photos or OCT were not good enough in the right eye. All the analyses including OCT, VF and refraction were obtained from the same eye as retinal vessel caliber measurement. Fundus photography was performed using Canon CF-60DSi Digital Mydriatic Fundus Camera with an attached Canon EOS-1Ds MK III SLR Digital Camera (Canon, Tokyo, Japan). CRAE and CRVE were calculated from papilla-centered images using the Integrative Vessel Analysis (IVAN) software V.1.3 (Department of Ophthalmology and Visual Science, University of Wisconsin, Madison, Wisconsin, USA) with Knudtson’s method, as previously described^18^. AVR was calculated as a ratio of CRAE and CRVE. Optical coherence tomography (OCT) measurements were obtained with a spectral/Fourier-domain OCT Cirrus HD-OCT 4000 device (software V.6.0.0; Carl Zeiss Meditec). The macular cube program of the OCT was used to measure macular parameters (central subfield thickness and macular thickness and volume). Mean RNFL thickness was measured along a circle concentric with the optic nerve head (ONH)^14^. Visual field examination was conducted using 24-2 Swedish Interactive Threshold Algorithm (SITA) of the Humphrey field analyzer (HFA). Visual field examination was included only if it was considered reliable: the rate of false positives less than 15% and the rate of fixation losses less than 20%. The age-adjusted mean deviation (MD) of the central visual field and age-adjusted pattern standard deviation (PSD) was documented.

### Statistical analyses

Prior to analyses, the data was inspected visually for skewness and kurtosis. The differences in group means between males and females were compared using Student's T-test in normally distributed variables. For variables with skewed distribution non-parametric Mann–Whitney's U-test was used. For analyses, the study population was first divided to sex-specific quintiles according to Hb levels (Fig. S1). For continuous variables one-way ANOVA and for the categorical variable Pearson's Chi-squared were used for Hb quintile comparisons. *P*-values < 0.05 were considered statistically significant. Linear regression models were used to assess associations between Hb levels and outcome variables. Logarithmic transformation of the variable was used for skewed variables. First, association was tested using simple linear regression (Model 1) and then by adjusted for smoking, refractive error and BP (Model 2). To account for further confounders the regression models were further adjusted for BMI, HOMA-IR, Matsuda index, hsCRP, LDL cholesterol, HDL cholesterol, triglycerides, and albumin levels (Model 3), and additionally for fellow vessel caliber, that is CRAE values were adjusted for CRVE values and vice versa (Model 4). All effect sizes (B) and 95% confidence intervals (CIs) were estimated using standardized variables. The level of statistical significance was set to 95%. The Hb quintile mean plots were created using the GraphPad Prism (version 9.3.1; GraphPad software LLC) and all statistical analyses were carried out with IBM SPSS Statistics for Windows, Version 28.0 (IBM Corp. Armonk, NY).

## Results

### Characteristics of the study population

There were 2319 (42.1% males, age 47 ± 1 yrs) subjects in the final study population. Characteristics of the study population in Hb quintiles are presented in Tables 1, 2, S1 and S2. Expected sex-specific differences were seen in the studied parameters (Table S2). Differences in several parameters were found between the Hb quintiles in both sexes (Tables 1, 2). Difference in smoking status was observed in females, the highest Hb quintile having the most current smokers, but no difference in smoking status between Hb quintiles was observed in males (Tables 1, 2). The highest Hb quintile had the highest BMI, the highest BP values and insulin resistance scores, the least favorable lipid profile, and the highest albumin levels in both sexes (Tables 1, 2). No difference in kidney function parameters or hsCRP were observed between the Hb quintiles for either sex (Tables 1, 2). The highest Hb quintile for males had the lowest urea concentration while no difference between the Hb quintiles was detected for females.

**Table 1:** Characteristics of the males of the study population in Hb quintiles.

	Q1	Q2	Q3	Q4	Q5	*P*
	M(SD)/n(%)	M(SD)/n(%)	M(SD)/n(%)	M(SD)/n(%)	M(SD)/n(%)	
No. of participants (n)	202	199	222	174	180	
Hb (g/L)	140.4 (3.0)	147.3 (1.4)	151.9 (1.4)	156.3 (1.1)	161.9 (2.5)	< 0.001
Smoking status (%)						0.366
Never smoker	35.6	33.2	31.5	30.5	35.0	
Past smoker	53.0	50.3	51.8	48.9	45.0	
Current smoker	11.4	16.6	16.7	20.7	20.0	
Weight (kg)	81.4 (12.7)	87.1 (14.1)	86.2 (13.8)	89.4 (15.4)	93.1 (16.3)	< 0.001
Height (m)	1.78 (0.06)	1.79 (0.06)	1.79 (0.07)	1.79 (0.06)	1.79 (0.07)	0.125
BMI (kg/m^2^)	25.8 (3.8)	27.2 (4.2)	27.0 (3.8)	27.9 (4.2)	29.1 (4.7)	< 0.001
SBP (mmHg)	127.2 (13.1)	129.2 (14.5)	130.8 (14.0)	131.5 (14.6)	134.9 (15.0)	< 0.001
DBP (mmHg)	84.1 (9.6)	85.1 (10.4)	86.7 (9.8)	88.1 (10.0)	90.7 (9.5)	< 0.001
HOMA-IR	1.9 (2.2)	2.5 (2.0)	2.8 (2.9)	3.2 (3.0)	3.8 (2.2)	< 0.001
Matsuda index	118.5 (63.5)	91.7 (57.5)	90.6 (54.5)	79.2 (51.8)	72.0 (44.6)	< 0.001
Total cholesterol (mmol/L)	5.3 (0.9)	5.6 (0.9)	5.6 (1.0)	5.5 (1.0)	5.6 (0.9)	0.008
LDL cholesterol (mmol/L)	3.5 (0.9)	3.7 (0.9)	3.7 (0.9)	3.7 (0.9)	3.8 (0.9)	< 0.001
HDL cholesterol (mmol/L)	1.5 (0.3)	1.4 (0.3)	1.4 (0.4)	1.3 (0.3)	1.3 (0.3)	< 0.001
Triglycerides (mmol/L)	1.2 (0.7)	1.5 (1.1)	1.5 (1.4)	1.5 (0.8)	1.7 (0.9)	< 0.001
hsCRP (mg/L)	1.4 (2.6)	1.4 (2.6)	1.3 (1.7)	1.2 (1.6)	1.6 (3.5)	0.544
Albumin (g/L)	45.3 (2.1)	46.0 (2.0)	45.8 (2.0)	46.0 (2.2)	46.4 (2.1)	< 0.001
eGFR (mL/min/1.73m^2^)	101.7 (9.0)	103.1 (8.0)	101.2 (9.4)	102.7 (8.2)	102.1 (9.4)	0.204
Urea (mmol/L)	6.2 (1.3)	6.1 (1.2)	5.9 (1.2)	6.0 (1.3)	5.8 (1.2)	0.049
Refractive error (D)	-0.9 (2.1)	-0.8 (2.3)	-0.8 (1.9)	-0.7 (2.4)	-0.9 (2.2)	0.904
CRAE (μm)	142.1 (13.4)	142.0 (12.9)	143.5 (13.5)	142.5 (12.7)	139.5 (14.9)	0.088
CRVE (μm)	216.7 (19.0)	219.5 (18.2)	222.2 (17.2)	221.4 (17.1)	222.6 (19.2)	0.016
AVR	0.7 (0.1)	0.6 (0.1)	0.6 (0.1)	0.6 (0.1)	0.6 (0.1)	< 0.001
Average RNFL th. (μm)	90.2 (9.8)	90.5 (9.8)	90.6 (11.5)	90.9 (9.7)	91.5 (9.1)	0.793
Central subfield th. (μm)	270.5 (19.2)	267.9 (20.1)	266.9 (23.5)	270.1 (22.1)	268.3 (20.4)	0.417
Macular th. (μm)	284.8 (16.5)	284.3 (14.3)	284.4 (13.1)	283.1 (14.8)	283.6 (12.8)	0.827
Macular V (mm^3^)	10.2 (0.6)	10.2 (0.5)	10.2 (0.5)	10.1 (0.5)	10.1 (0.5)	0.854
MD (dB)	-0.1 (1.6)	-0.1 (1.7)	0.0 (1.5)	0.0 (1.4)	-0.2 (1.8)	0.642
PSD	1.76 (1.05)	1.78 (1.32)	1.67 (0.72)	1.73 (0.78)	1.91 (1.06)	0.206

The values are mean with (SD) or percentages. *P* is given for comparison over the Hb quintiles in one-way ANOVA and for the categorical variables Pearson’s Chi-squared were used for Hb quintile comparisons.

Q, quintile; M, Mean; SD, standard deviation; No, number of; Hb, hemoglobin; BMI, body mass index; SBP, systolic blood pressure; DBP, diastolic blood pressure, HbA1c, glycated hemoglobin; HOMA-IR, homeostatic model assessment for insulin resistance; Matsuda index, Matsuda index for whole-body insulin sensitivity; LDL, low-density lipoprotein. HDL, high-density lipoprotein; hsCRP, high sensitivity C-reactive protein; eGFR, estimated glomerular filtration rate; CRAE, central retinal artery equivalent; CRVE, central retinal vein equivalent; AVR, arteriovenous ratio; RNFL, retinal nerve fiber layer; th, thickness; V, volume; MD, mean deviation; PSD, pattern standard deviation.

**Table 2:** Characteristics of the females of the study population in Hb quintiles.

	Q1	Q2	Q3	Q4	Q5	*P*
	M(SD)/n(%)	M(SD)/n(%)	M(SD)/n(%)	M(SD)/n(%)	M(SD)/n(%)	
No. of participants (n)	290	264	263	300	225	
Hb (g/L)	123.5 (2.8)	130.1 (1.4)	134.6 (1.1)	139.4 (1.7)	146.9 (3.4)	< 0.001
Smoking status (%)						< 0.001
Never smoker	39.3	38.6	38.4	39.0	38.2	
Past smoker	50.0	53.0	50.2	46.0	37.3	
Current smoker	10.7	8.3	11.4	15.0	24.4	
Weight (kg)	67.4 (13.7)	70.1 (13.0)	71.0 (13.2)	73.8 (15.5)	77.9 (16.3)	< 0.001
Height (m)	1.64 (0.06)	1.65 (0.05)	1.65 (0.06)	1.65 (0.05)	1.65 (0.06)	0.182
BMI (kg/m^2^)	25.0 (4.7)	25.6 (4.6)	26.1 (4.7)	27.1 (5.4)	28.6 (5.8)	< 0.001
SBP (mmHg)	117.9 (14.3)	119.0 (14.1)	120.4 (14.3)	122.2 (15.7)	124.7 (16.9)	< 0.001
DBP (mmHg)	80.1 (9.6)	81.3 (9.7)	82.2 (9.6)	84.8 (10.6)	86.9 (11.5)	< 0.001
HOMA-IR	2.2 (4.6)	1.9 (1.5)	1.9 (1.2)	2.2 (2.0)	3.4 (5.0)	< 0.001
Matsuda index	128.5 (64.7)	120.2 (61.1)	112.7 (66.6)	101.5 (48.4)	90.2 (56.2)	< 0.001
Total cholesterol (mmol/L)	5.1 (0.8)	5.2 (0.8)	5.1 (0.8)	5.3 (0.9)	5.2 (1.0)	0.019
LDL cholesterol (mmol/L)	3.1 (0.8)	3.2 (0.8)	3.2 (0.8)	3.4 (0.9)	3.4 (1.0)	< 0.001
HDL cholesterol (mmol/L)	1.7 (0.4)	1.7 (0.4)	1.7 (0.4)	1.6 (0.4)	1.5 (0.3)	< 0.001
Triglycerides (mmol/L)	0.9 (0.4)	1.0 (0.5)	1.0 (0.5)	1.1 (0.6)	1.2 (0.5)	< 0.001
hsCRP (mg/L)	1.5 (3.0)	1.5 (3.0)	1.6 (4.6)	1.7 (2.5)	2.2 (3.7)	0.150
Albumin (g/L)	43.7 (2.0)	43.9 (2.2)	44.2 (2.2)	44.7 (2.3)	44.9 (2.2)	< 0.001
eGFR (mL/min/1.73m^2^)	100.2 (10.4)	99.6 (9.4)	99.4 (10.6)	99.4 (10.8)	100.0 (10.7)	0.862
Urea (mmol/L)	4.9 (1.2)	5.0 (1.1)	4.9 (1.1)	5.1 (1.2)	5.0 (1.0)	0.305
Refractive error (D)	-1.2 (2.4)	-1.3 (2.5)	− 1.4 (2.5)	− 1.3 (2.6)	− 1.5 (2.7)	0.816
CRAE (μm)	140.9 (13.9)	140.1 (14.7)	141.6 (15.0)	139.8 (14.4)	139.9 (13.8)	0.578
CRVE (μm)	215.2 (19.2)	213.5 (20.6)	217.8 (19.6)	216.4 (19.0)	221.0 (19.8)	0.001
AVR	0.7 (0.1)	0.7 (0.1)	0.7 (0.1)	0.6 (0.1)	0.6 (0.1)	< 0.001
Average RNFL th. (μm)	91.7 (9.0)	91.3 (8.3)	91.6 (9.0)	91.6 (10.4)	91.1 (11.2)	0.959
Central subfield th. (μm)	253.8 (20.0)	255.1 (22.4)	256.8 (20.8)	255.9 (20.3)	256.7 (19.9)	0.442
Macular th. (μm)	279.2 (14.8)	279.6 (13.4)	281.4 (14.1)	281.1 (13.7)	279.1 (12.9)	0.166
Macular V (mm^3^)	10.0 (0.5)	10.0 (0.5)	10.1 (0.5)	10.0 (0.5)	10.0 (0.5)	0.168
MD (dB)	0.0 (1.9)	0.1 (1.6)	0.0 (2.1)	− 0.1 (2.1)	− 0.2 (2.6)	0.656
PSD	1.82 (1.28)	1.71 (1.14)	1.82 (0.94)	1.84 (1.35)	1.82 (1.00)	0.732

The values are mean with (SD) or percentages. *P* is given for comparison over the Hb quintiles in one-way ANOVA and for the categorical variables Pearson’s Chi-squared were used for Hb quintile comparisons.

Q, quintile; M, Mean; SD, standard deviation; No, number of; Hb, hemoglobin; BMI, body mass index; SBP, systolic blood pressure; DBP, diastolic blood pressure, HbA1c, glycated hemoglobin; HOMA-IR, homeostatic model assessment for insulin resistance; Matsuda index, Matsuda index for whole-body insulin sensitivity; LDL, low-density lipoprotein. HDL, high-density lipoprotein; hsCRP, high sensitivity C-reactive protein; eGFR, estimated glomerular filtration rate; CRAE, central retinal artery equivalent; CRVE, central retinal vein equivalent; AVR, arteriovenous ratio; RNFL, retinal nerve fiber layer; th, thickness; V, volume; MD, mean deviation; PSD, pattern standard deviation.

### Differences in retinal parameters in sex-specific Hb quintiles

**Figure 1:**
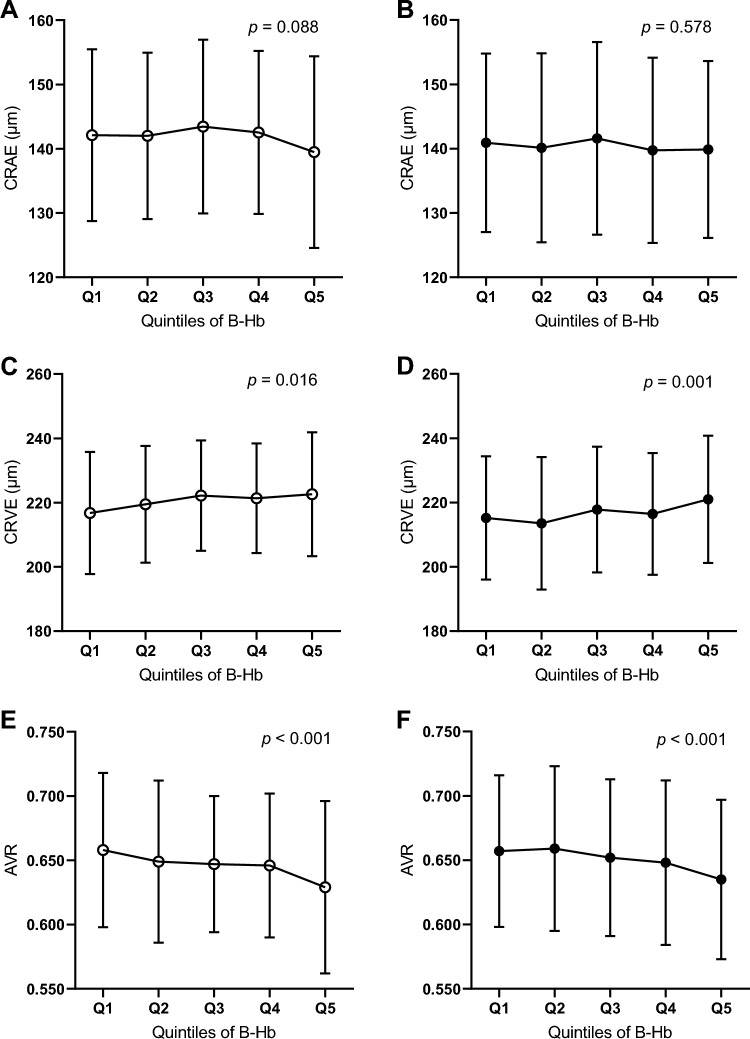
Retinal vascular caliber parameters in sex-specific Hb quintiles. CRAE (**A**, **B**), CRVE (**C**, **D**) and AVR (**E**, **F**) in Hb quintiles for males (white dot; **A, C, E**) and females (black dot; **B, D, F**). The values are mean with (SD). *P* is given for comparison over the Hb quintiles in one-way ANOVA. Q, quintile; B-Hb, blood hemoglobin; CRAE, central retinal artery equivalent; CRVE, central retinal vein equivalent; AVR, arteriovenous ratio.

Vascular and neural parameters of the retina were analyzed in sex-specific Hb quintiles (Figs. 1, S2, S3 and Tables S1, S2). For either sex, no difference in CRAE was observed between the Hb quintiles (Fig. 1A,B, Tables S1, S2). Differences in CRVE and AVR were observed between Hb quintiles for both sexes with the highest Hb quintiles having the highest CRVE and the lowest AVR. No difference in RNFL thickness or any studied macular or visual field parameters were observed between the Hb quintiles (Figs. S2, S3 and Table S3).

### Linear regression models for association of Hb levels with ophthalmological and renal function parameters

**Figure 2:**
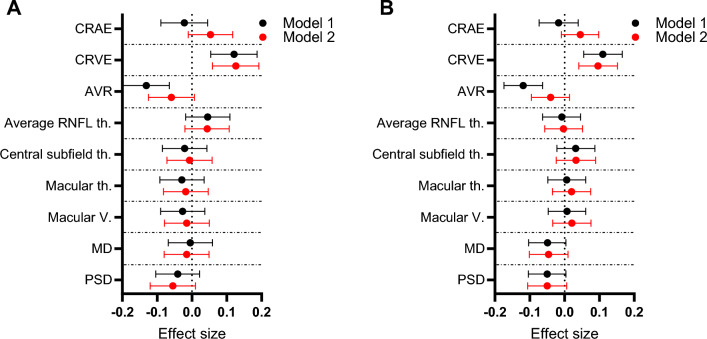
Sex-specific effect sizes for association of Hb levels with ophthalmological parameters. Forest plot representing the effect sizes and their 95% CIs for Hb levels and ophthalmological parameters in males (**A**) and females (**B**). The CIs and effect sizes represent change in standardized variables. Model 1 (presented in black) represents unadjusted linear regression. Model 2 (presented in red) is a linear regression model adjusted for smoking, BP and refractive error. CRAE, central retinal artery equivalent; CRVE, central retinal vein equivalent; AVR, arteriovenous ratio; RNFL, retinal nerve fiber layer; th, thickness; V, volume; MD, mean deviation; PSD, pattern standard deviation.

To evaluate the independent associations of Hb levels and vascular and neural parameters, linear regression models were used (Fig. 2, Table S3). For both sexes, Hb levels associated positively with CRVE (*B*_males_ = 0.121 [0.054; 0.188], *B*_females_ = 0.110 [0.055; 0.166]) in an unadjusted regression model (Fig. 2A,B, Model 1, presented in black) and when adjusted for smoking, BP and refractive error (Fig. 2A,B, Model 2, presented in red) (*B*_males_ = 0.126 [0.059; 0.192], *B*_females_ = 0.096 [0.041; 0.152]) (Fig. 2A,B, Table S3). No association of Hb levels with CRAE was observed, whereas for AVR there was a negative association with Hb levels for both sexes (*B*_males_ = − 0.131 [− 0.198; − 0.065], *B*_females_ = − 0.119 [− 0.174; − 0.063]) in Model 1 but not in Model 2 (Fig. 2A,B, Table S3). No associations of Hb levels with RNFL thickness, macular or visual field parameters were observed in either model or sex (Fig. 2A,B, Table S3).

Besides the retina, microvascular alterations in systemic diseases are typically manifested in the kidney. We therefore also analyzed the associations of Hb levels with kidney function parameters; serum albumin and urea, and eGFR, available for the participants of the NFBC Eye Study. There was a positive association between Hb levels and albumin in both sexes (*B*_males_ = 0.151 [0.090; 0.212], (*B*_females_ = 0.208 [0.153; 0.259]), and a weak negative association between Hb levels and urea in males (*B* = − 0.090 [− 0.156; − 0.030]), while no association between Hb levels and eGFR was detected (Table S4). No associations between CRVE and any of the kidney parameters were found (Table S5).

### Association of Hb levels with retinal vascular parameters in multivariable linear regression models

**Figure 3:**
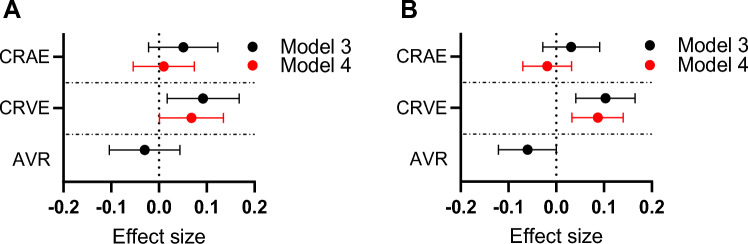
Sex-specific effect sizes for association of Hb levels with retinal vascular parameters in multivariable linear regression models. Forest plot representing the effect sizes and their 95% CIs for Hb levels and selected ophthalmological parameters in males (**A**) and females (**B**). The CIs and effect sizes represent change in standardized variables. Model 3 (presented in black) is adjusted for smoking, blood pressure, refractive error, BMI, HOMA-IR, Matsuda index, hsCRP, LDL cholesterol, HDL cholesterol, triglycerides, and albumin levels, and Model 4 (presented in red), additionally for fellow vessel caliber. CRAE, central retinal artery equivalent; CRVE, central retinal vein equivalent; AVR, arteriovenous ratio.

As the retinal vascular parameters showed associations with Hb levels in the initial analyses, we therefore evaluated their association in multivariable linear regression models, and further, in more optimally adjusted linear regression models (Fig. 3, Table S6). Hb levels associated with CRVE in both sexes (*B*_males_ = 0.092 [0.017; 0.168], *B*_females_ = 0.103 [0.041; 0.165]) in Model 3 (presented in black), adjusted for smoking, BP, refractive error, BMI, HOMA-IR, Matsuda index, hsCRP, LDL cholesterol, HDL cholesterol, triglycerides, and albumin levels, and in Model 4 (presented in red), (*B*_males_ = 0.068 [0.001; 0.135], *B*_females_ = 0.087 [0.033; 0.140]), adjusted additionally for fellow vessel caliber (CRVE or CRAE) (Fig. 3, Table S6). No associations of Hb levels with either CRAE or AVR were observed using Model 3, nor in the case of CRAE, in Model 4 (Fig. 3, Table S6).

## Discussion

Here, we showed that Hb levels within its reference interval associate positively and specifically with CRVE in a middle-aged Finnish study population in both sexes. The association was independent of cardiometabolic factors that we have recently shown to associate with Hb levels^7^. Lower Hb levels were associated with higher expression of HIF target genes, suggesting that Hb levels via tissue oxygenation regulate the HIF pathway^7^, which is a powerful regulator of vasculature and energy metabolism. As retinal tissue consumes vast amounts of oxygen constantly, and Hb levels can be used as a surrogate marker for tissue oxygenation, we wanted to explore the association between Hb levels and vascular and neural parameters.

In the quintile analyses the lowest Hb quintile had the lowest CRVE, but no differences were observed in other retinal or visual field parameters. Also, lower Hb levels appeared favorable in terms of established CVD risk factors and other metabolic parameters, which is in line with previously published results by our group^7–9^. Since we found several differences between the lowest and the highest Hb quintiles, variables with significant differences over quintiles were used as covariates in linear regression models to avoid the effect of confounding factors on vascular or neural parameters or Hb. Additionally, hsCRP was included as a covariate since it has earlier been shown to affect retinal vascular caliber^20^. Total cholesterol levels were omitted as a covariate from the linear models due to strong collinearity with LDL cholesterol and HDL cholesterol levels. We believe that the selection of these covariates resulted in the most reliable analysis but cannot rule out the contribution of other covariates.

CRVE, but not CRAE or AVR, was associated positively with Hb levels in linear regression models also when adjusted for multiple covariates and fellow vessel caliber. To the best of our knowledge, a positive association between Hb levels and CRVE has previously been shown in Western populations exclusively in elderly people, with mean age over 60 years^11–13^. In addition to CRVE, CRAE was found to be positively associated with Hb levels but to a lesser extent than CRVE in earlier studies^11,12^. However, this finding was not replicated in all studies^13^, and Hb levels were inversely associated with CRAE after adjustment for fellow vessel caliber^12^. This discrepancy potentially rises from the study population where people with extremely low and high Hb levels were included^11,12^. Also, the reported CRAE varied from 140 to 190 μm, this fluctuation potentially originating from the use of different formulas for measuring the vascular caliber^11–13^. Also, laboratory parameters used as covariates in multivariable linear regression models varied in the earlier published studies^11–13^.

Based on the presented results, CRVE varies by approximately 8 μm (4%) in females within the reference interval of Hb levels, the observation being less prominent in males. In regards of clinical significance, Hb levels outweighted the relative importance of insulin resistance indices and serum lipids and inflammatory parameters on regulating CRVE diameter in our models in females, respectively. For now, further clinical applicability of these data remain to be elucidated. Both higher Hb levels and wider CRVE have been shown to be associated with cardiovascular diseases (e.g., hypertension, coronary artery disease, stroke)^4,6^. It seems that Hb levels have a parallel effect with metabolic factors, smoking, and inflammation on CRVE. It remains to be established whether Hb levels associate with microvascular diameter in other tissues.

The physiological and biochemical mechanisms underlying the associations of Hb levels and retinal vascular caliber remain unknown. Blood viscosity and factors related to tissue oxygenation, chronic hypoxia, and nitric oxide mediated vasodilation have been suggested as possible mediators of this association^11–13^. Although not directly measured in this study, we hypothesize that Hb levels, via tissue oxygenation, regulate blood vessel caliber in a HIF-dependent manner, the effect being more prominent on the venular side, where O_2_ pressure is lower and the vascular structure is more delicate. Of the HIF targets, overexpression of the genes coding for angiopoietin-1 and its receptor Tie-2, and apelin and its receptor APJ have been associated in earlier studies to larger blood vessel diameters ^21–24^.

Associations between RVD and OCT parameters was recently published in the NFBC Eye Study^19^. However, we did not find an association of the OCT parameters with Hb levels. This may simply be due to anatomical positioning where Hb/oxygen is in direct contact with vasculature compared to the more distant neuronal layers. Therefore, the vasculature represents the primary contact point for hypoxia. Further studies are needed to evaluate the potential associations of Hb levels with neuroretinal parameters in older people.

Limitations of this study are lack of longitudinal data and iron metabolism parameters. We have not carried out a correction for multiple testing in the models adjusted for multiple covariates. Possible measurement errors in ophthalmological variables, such as intergrader variability, cardiac cycle, and imaging technique factors, might also have influenced our results, as previously discussed^25^, but not more than in other studies measuring RVDs. In turn, the large number of study subjects and randomization of the screening and control groups can be considered as desirable. A major strength of this study is the population’s homogeneity age wise, and ethnically, and by similar altitude and environmental exposure. Although adjusted for multiple covariates, residual confounding effect needs to be considered as an explanatory factor for the observed association.

In summary, Hb levels within reference interval were associated positively with CRVE, and the association was in parallel with cardiometabolic risk factors but independent of them. Therefore, Hb levels should be considered when assessing retinal vascular caliber.

### Supplementary Information

Supplementary Information.

## Data Availability

NFBC data is available from the University of Oulu, Infrastructure for Population Studies. Permission to use the data can be applied for research purposes via an electronic material request portal. In the use of data, we follow the EU general data protection regulation (679/2016) and Finnish Data Protection Act. Please, contact NFBC project center (NFBCprojectcenter(at)oulu.fi) and visit the cohort website for more information (University of Oulu, 1966).
