# Supplementary material for: Hemoglobin levels are associated with retinal vascular caliber in a middle-aged birth cohort
Source: Sci Rep. 2024 Apr 20;14:9092. doi: 10.1038/s41598-024-59688-y (PMC11032340; doi:10.1038/s41598-024-59688-y)
Supplement: Supplementary file 1 — Supplementary Information. [file 41598_2024_59688_MOESM1_ESM.pdf]

Supplementary information

Hemoglobin levels are associated with retinal vascular caliber in a middle-aged birth cohort

Running head: Hemoglobin and vascular caliber

Samuli Sakko[1], Mikko Karpale[1], Joona Tapio[1], Iina Leppänen[2], Oona Ahokas[2], Ville Saarela[2], M. Johanna Liinamaa[2*], Peppi Koivunen[1*]

[1]Biocenter Oulu and Faculty of Biochemistry and Molecular Medicine, Oulu Center for Cell-Matrix Research, University of Oulu, P.O. Box 5400, FIN-90014 Oulu, Finland.

[2]Department of Ophthalmology, Oulu University Hospital, Medical Research Center Oulu and Research Unit of Clinical Medicine, University of Oulu, Oulu, Finland.

*To whom correspondence should be addressed:

Peppi Koivunen, email: peppi.koivunen@oulu.fi,

Johanna Liinamaa, email: johanna.liinamaa@oulu.fi

Table S1. Exact number of participants for each studied parameter in quintile analyses presented in Table 1. Q, quintile; Hb, hemoglobin; BMI, body mass index; SBP, systolic blood pressure; DBP, diastolic blood pressure, HbA1c, glycated hemoglobin; HOMA-IR, homeostatic model assessment for insulin resistance; Matsuda index, Matsuda index for whole-body insulin sensitivity; LDL, low-density lipoprotein. HDL, high-density lipoprotein; hsCRP, high sensitivity C-reactive protein; eGFR, estimated glomerular filtration rate; CRAE, central retinal artery equivalent; CRVE, central retinal vein equivalent; AVR, arteriovenous ratio; RNFL, retinal nerve fiber layer; th, thickness; V, volume; MD, mean deviation; PSD, pattern standard deviation.

	Males						Females				
	Q1	Q2	Q3	Q4	Q5		Q1	Q2	Q3	Q4	Q5
Hb (g/L)	202	199	222	174	180		290	264	263	300	225
Smoking status	202	199	222	174	180		290	264	263	300	225
Never smoker	72	66	70	53	63		114	102	101	117	86
Past smoker	107	100	115	85	81		145	140	132	138	84
Current smoker	23	33	37	36	36		31	22	30	45	55
Weight (kg)	201	199	222	174	180		290	264	263	300	225
Height (m)	201	199	222	174	180		290	264	263	300	225
BMI (kg/m^2)	201	199	222	174	180		290	264	263	300	225
SBP (mmHg)	202	199	222	174	180		290	264	263	300	225
DBP (mmHg)	202	199	222	174	180		290	264	263	300	225
HOMA-IR	194	193	216	170	177		278	255	257	298	223
Matsuda index	178	176	191	145	152		251	230	233	269	190
Total cholesterol (mmol/L)	202	199	221	174	180		290	264	263	300	225
HDL cholesterol (mmol/L)	202	199	222	174	180		290	264	263	300	225
LDL cholesterol (mmol/L)	202	199	222	174	180		290	264	263	300	225
Triglycerides (mmol/L)	202	199	222	174	180		290	264	263	300	225
hsCRP (mg/L)	201	198	221	172	178		288	264	262	299	224
Albumin (g/L)	202	199	222	174	180		290	264	263	300	225
eGFR (ml/min/1.73m^2)	200	199	220	173	179		290	263	263	299	225
Urea (mmol/L)	202	199	222	174	180		290	264	263	300	225
Refractive error (D)	202	199	222	174	180		290	264	263	300	225
CRAE (µm)	177	166	193	162	160		269	240	239	282	205
CRVE (µm)	177	166	193	162	160		269	240	239	282	205
AVR	177	166	193	162	160		269	240	239	282	205
Average RNFL th. (µm)	197	192	215	171	177		285	258	255	290	217
Central subfield th. (µm)	197	192	215	171	177		285	258	255	290	217
Macular th. (µm)	197	192	215	171	177		285	258	255	290	217
Macular V (mm^3)	197	192	215	171	177		285	258	255	290	217
MD (dB)	201	195	221	173	179		290	264	260	300	223
PSD (dB)	201	195	221	173	179		290	264	260	300	223

Table S2. Characteristics of males and females of the study population. M, Mean; SD, standard deviation; Mdn, median; CI, confidence interval; Hb, hemoglobin; BMI, body mass index; SBP, systolic blood pressure; DBP, diastolic blood pressure, HbA1c, glycated hemoglobin; HOMA-IR, homeostatic model assessment for insulin resistance; Matsuda index, Matsuda index for whole-body insulin sensitivity; LDL, low-density lipoprotein. HDL, high-density lipoprotein; hsCRP, high sensitivity C-reactive protein; eGFR, estimated glomerular filtration rate; CRAE, central retinal artery equivalent; CRVE, central retinal vein equivalent; AVR, arteriovenous ratio; RNFL, retinal nerve fiber layer; th, thickness; V, volume; MD, mean deviation; PSD, pattern standard deviation.

	Males		Females		
	N	M (SD)/ Mdn (CI)	N	M (SD)/ Mdn (CI)	*P*
Age (yrs)	971	47.3 (0.9)	1340	47.3 (0.9)	
Hb (g/L)	977	151.2 (7.6)	1342	134.4 (8.1)	<0.001
Weight (kg)	976	87.2 (14.9)	1342	71.8 (14.8)	<0.001
Height (m)	976	1.79 (0.06)	1342	1.65 (0.06)	<0.001
BMI (kg/m^2)	976	27.3 (4.3)	1342	26.4 (5.2)	<0.001
SBP (mmHg)	977	130.6 (14.4)	1342	120.7 (15.2)	<0.001
DBP (mmHg)	977	86.8 (10.1)	1342	82.9 (10.4)	<0.001
HOMA-IR*	950	2.1 (0.3 to 26.0)	1311	1.6 (0.4 to 58.2)	<0.001
Matsuda index*	842	77.5 (8.9 to 440.7)	1173	101.3 (14.2 to 522.9)	<0.001
Total cholesterol (mmol/L)	976	5.5 (0.9)	1342	5.2 (0.9)	<0.001
LDL cholesterol (mmol/L)	977	3.7 (0.9)	1342	3.3 (0.9)	<0.001
HDL cholesterol (mmol/L)	977	1.4 (0.3)	1342	1.7 (0.4)	<0.001
Triglycerides* (mmol/L)	977	1.2 (0.3 to 16.6)	1342	0.9 (0.3 to 4.9)	<0.001
hsCRP* (mg/L)	970	0.7 (0.2 to 42.4)	1337	0.8 (0.2 to 69.0)	0.279
Albumin (g/L)	977	45.9 (2.1)	1342	44.3 (2.2)	<0.001
eGFR (ml/min/1.73m^2)	971	102.1 (8.8)	1340	99.7 (10.4)	<0.001
Urea (mmol/L)	977	6.0 (1.27)	1342	5.0 (1.13)	<0.001
Refractive error* (D)	977	-0.3 (-16.3 to 10.3)	1342	-0.5 (-12.6 to 7.1)	<0.001
CRAE (μm)	858	142.0 (13.5)	1235	140.5 (14.4)	0.014
CRVE (μm)	858	220.5 (18.2)	1235	216.6 (19.7)	<0.001
AVR	858	0.65 (0.06)	1235	0.65 (0.06)	0.083
Average RNFL th. (μm)	952	90.7 (10.0)	1305	91.5 (9.6)	0.071
Central subfield th. (μm)	952	268.7 (21.1)	1305	255.6 (20.7)	<0.001
Macular th. (μm)	952	284.1 (14.3)	1305	280.1 (13.9)	<0.001
Macular V (mm^3)	952	10.2 (0.51)	1305	10.0 (0.5)	<0.001
MD* (dB)	969	0.1 (-15.6 to 2.4)	1337	0.3 (-32.8 to 8.9)	0.101
PSD* (dB)	969	1.54 (0.92 to 14.71)	1337	1.54 (0.88 to 15.92)	0.457

* Values as median for non-normally distributed variables

Table S3. Number of participants and exact values for sex-specific linear regression models of Hb levels with ophthalmological parameters presented in Figure 2. The CIs and effect sizes represent changes in standardized variables. Model 1 represents unadjusted linear regression. Model 2 is a linear regression model adjusted for smoking, bp and refractive error. N, number of participants in the analyses; B, effect size, CIL, confidence interval lower limit, CIU; confidence interval upper limit; CRAE, central retinal artery equivalent; CRVE, central retinal vein equivalent; AVR, arteriovenous ratio; RNFL, retinal nerve fiber layer; th, thickness; V, volume; MD, mean deviation; PSD, pattern standard deviation.

	Males					Females				
Model 1	N	B	CIL	CIU	*P*	N	B	CIL	CIU	*P*
CRAE	858	-0.022	-0.089	0.045	0.527	1235	-0.017	-0.073	0.039	0.542
CRVE	858	0.121	0.054	0.187	<0.001	1235	0.110	0.055	0.166	<0.001
AVR	858	-0.131	-0.198	-0.065	<0.001	1235	-0.119	-0.174	-0.063	<0.001
Average RNFL th.	952	0.045	-0.018	0.109	0.164	1305	-0.008	-0.063	0.046	0.765
Central subfield th.	952	-0.021	-0.085	0.043	0.518	1305	0.032	-0.022	0.087	0.245
Macular th.	952	-0.029	-0.092	0.035	0.376	1305	0.006	-0.048	0.061	0.823
Macular V	952	-0.027	-0.090	0.037	0.408	1305	0.007	-0.047	0.061	0.804
MD	969	-0.005	-0.068	0.059	0.886	1337	-0.049	-0.103	0.004	0.072
PSD	969	-0.041	-0.104	0.022	0.200	1337	-0.050	-0.104	0.004	0.067
Model 2	N	B	CIL	CIU	*P*	N	B	CIL	CIU	*P*
CRAE	858	0.053	-0.011	0.117	0.104	1235	0.045	-0.009	0.098	0.099
CRVE	858	0.126	0.059	0.192	<0.001	1235	0.096	0.041	0.152	<0.001
AVR	858	-0.059	-0.125	0.007	0.080	1235	-0.040	-0.095	0.014	0.144
Average RNFL th.	952	0.044	-0.020	0.107	0.180	1305	-0.003	-0.057	0.052	0.922
Central subfield th.	952	-0.007	-0.072	0.058	0.830	1305	0.033	-0.024	0.089	0.256
Macular th.	952	-0.018	-0.082	0.047	0.587	1305	0.020	-0.035	0.075	0.478
Macular V	952	-0.015	-0.079	0.050	0.650	1305	0.021	-0.034	0.076	0.456
MD	969	-0.015	-0.080	0.049	0.642	1337	-0.046	-0.101	0.010	0.107
PSD	969	-0.055	-0.120	0.010	0.096	1337	-0.050	-0.106	0.006	0.081

Table S4. Unadjusted sex-specific linear associations of kidney function parameters with Hb levels. The CIs and effect sizes represent changes in standardized variables. Hb, hemoglobin; N, number of participants in the analyses; B, effect size; CIL, confidence interval lower limit, CIU; confidence interval upper limit; eGFR, estimated glomerular filtration rate. Hb levels were used as a determinant.

	Males					Females				
Hb	N	B	CIL	CIU	*P*	N	B	CIL	CIU	*P*
Albumin	977	0.151	0.090	0.212	<0.001	1342	0.208	0.153	0.259	<0.001
eGFR	971	0.010	-0.051	0.072	0.746	1340	-0.017	-0.070	0.036	0.530
Urea	977	-0.090	-0.156	-0.030	0.005	1342	0.020	-0.033	0.067	0.471

Table S5. Unadjusted sex-specific linear associations of kidney function parameters with CRVE levels. The CIs and effect sizes represent changes in standardized variables. N, number of participants in the analyses; B, effect size; CIL, confidence interval lower limit, CIU; confidence interval upper limit; eGFR, estimated glomerular filtration rate. Albumin, eGFR and urea were used as determinants.

	Males					Females				
CRVE	N	B	CIL	CIU	*P*	N	B	CIL	CIU	*P*
Albumin	858	0.027	-0.040	0.094	0.428	1235	-0.054	-0.109	0.001	0.056
eGFR	858	0.042	-0.025	0.109	0.220	1235	-0.034	-0.089	0.022	0.233
Urea	858	-0.020	-0.086	0.046	0.552	1235	-0.025	-0.080	0.030	0.371

Table S6. Number of participants and exact values for sex-specific effect sizes for association of Hb levels with selected ophthalmological parameters in multivariable linear regression models presented in Figure 3. The CIs and effect sizes represent changes in standardized variables. Model 3 is adjusted for smoking, blood pressure, refractive error, BMI, HOMA-IR, Matsuda index, hsCRP, LDL cholesterol, HDL cholesterol, triglycerides, and albumin levels, and Model 4 additionally for fellow vessel caliber. N, number of participants in the analyses; B, effect size, CIL, confidence interval lower limit, CIU; confidence interval upper limit; CRAE, central retinal artery equivalent; CRVE, central retinal vein equivalent; AVR, arteriovenous ratio.

	Males					Females				
Model 3	N	B	CIL	CIU	*P*	N	B	CIL	CIU	*P*
CRAE	720	0.051	-0.022	0.123	0.171	1049	0.031	-0.028	0.091	0.303
CRVE	720	0.092	0.017	0.168	0.017	1049	0.103	0.041	0.165	0.001
AVR	720	-0.030	-0.104	0.044	0.426	1049	-0.060	-0.121	0.000	0.051
Model 4	N	B	CIL	CIU	*P*	N	B	CIL	CIU	*P*
CRAE	720	0.010	-0.054	0.074	0.759	1049	-0.019	-0.070	0.032	0.466
CRVE	720	0.068	0.001	0.135	0.047	1049	0.087	0.033	0.140	0.002

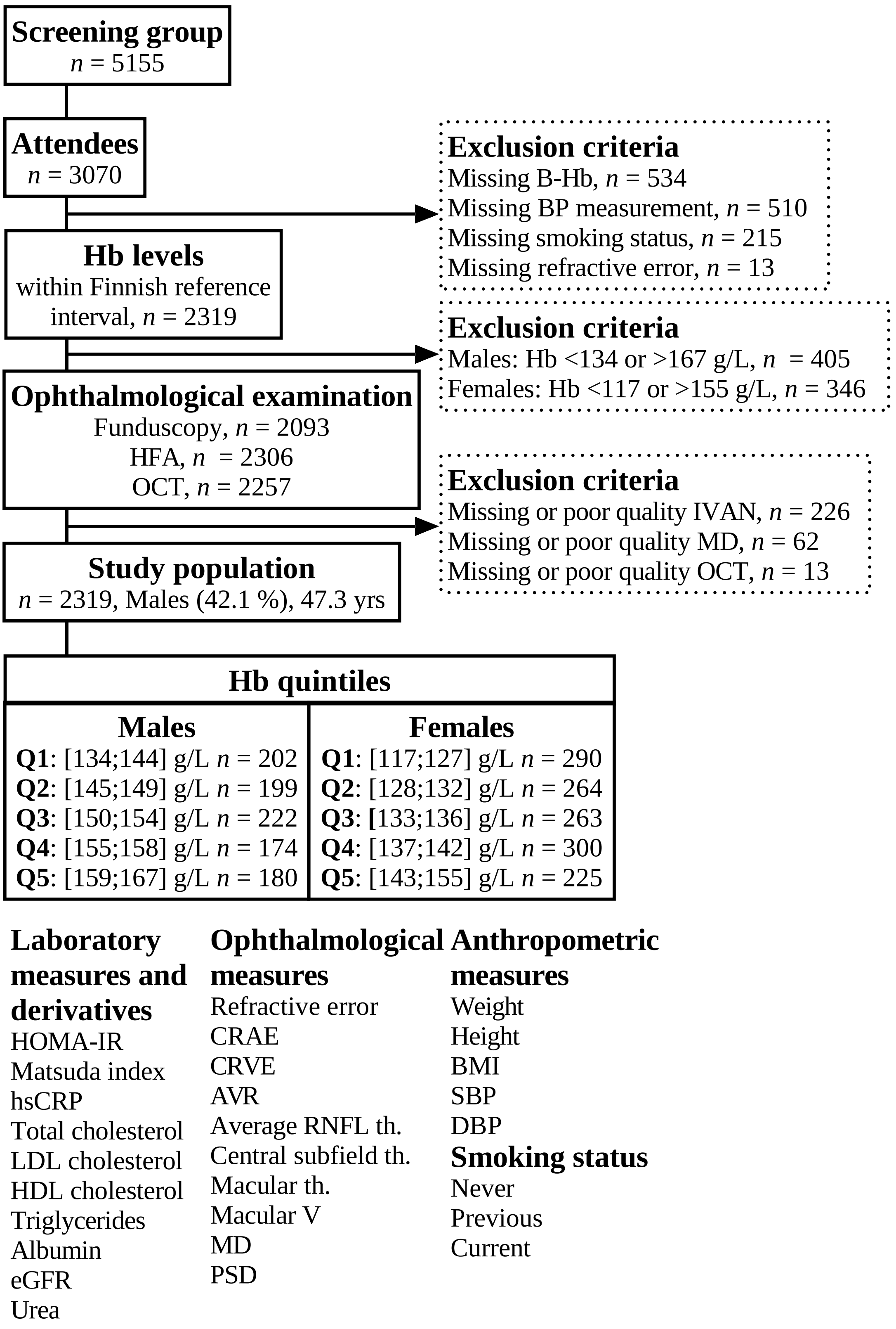

Figure S1. Flow chart representing the study population and analyses done. B-Hb, blood hemoglobin; bp, blood pressure; HFA, Humphrey field analyzer; MD, mean deviation; IVAN, Integrative Vessel Analysis; OCT, optical coherence tomography; Q, Hb quintile; BMI, body mass

index; SBP, systolic blood pressure; DBP, diastolic blood pressure, HbA1c, glycated hemoglobin; HOMA-IR, homeostatic model assessment for insulin resistance; Matsuda index, Matsuda index for whole-body insulin sensitivity; LDL, low-density lipoprotein. HDL, high-density lipoprotein; hsCRP, high sensitivity C-reactive protein; eGFR, estimated glomerular filtration rate; CRAE, central retinal artery equivalent; CRVE, central retinal vein equivalent; AVR, arteriovenous ratio; RNFL, retinal nerve fiber layer; th, thickness; V, volume; PSD, pattern standard deviation.

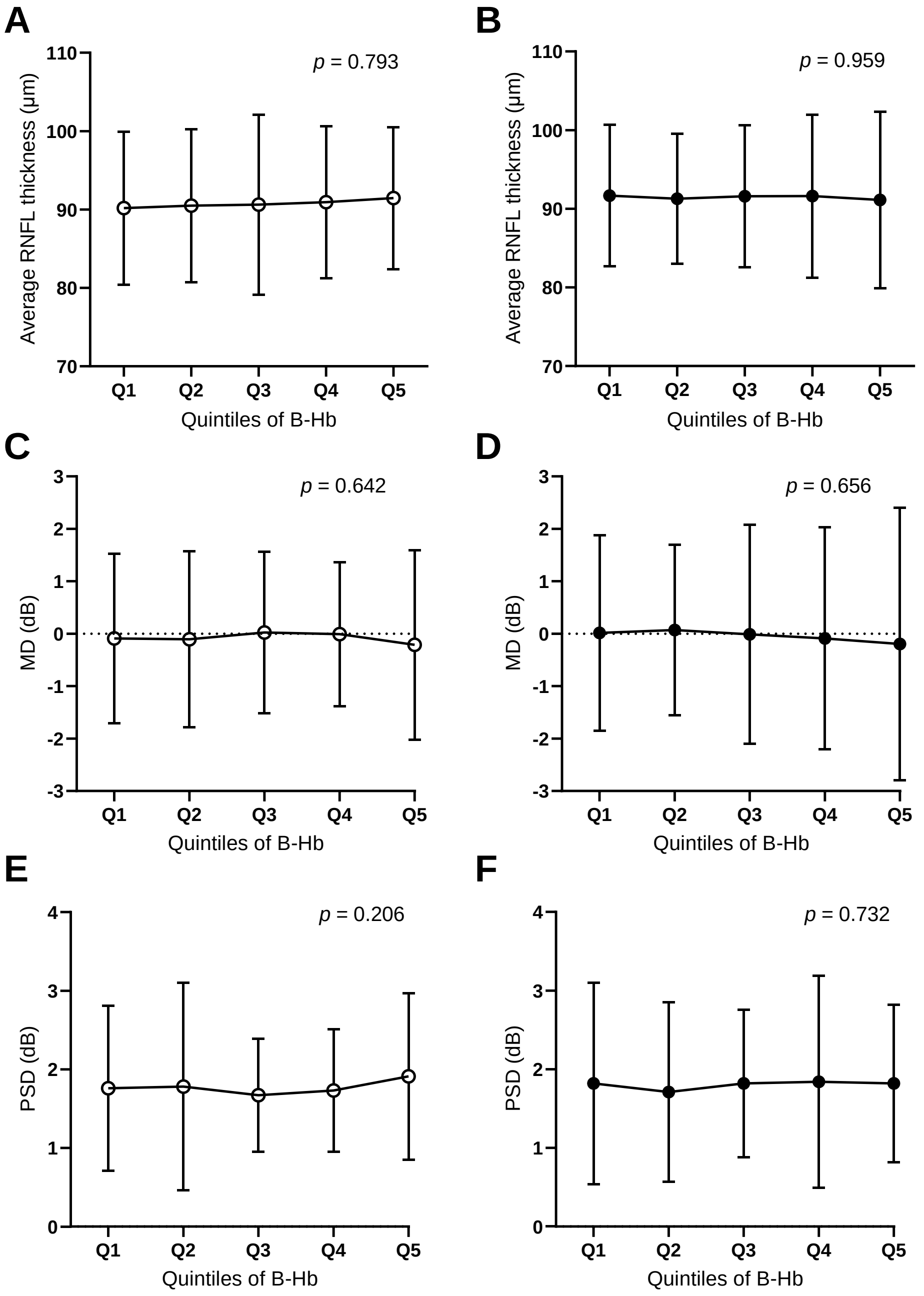

Figure S2. Average retinal nerve fiber layer thickness and mean deviation of visual field in sex-specific Hb quintiles. Average RNFL thickness (**A**, **B**) and MD (**C**, **D**) in Hb quintiles for males (white dot; **A, C**) and females (black dot; **B, D**). The values are mean with (SD) or percentages. *P* is given for comparison over the Hb quintiles in one-way ANOVA. Q, quintile; B-Hb, blood hemoglobin; RNFL, retinal nerve fiber layer; MD, mean deviation; PSD, pattern standard deviation.

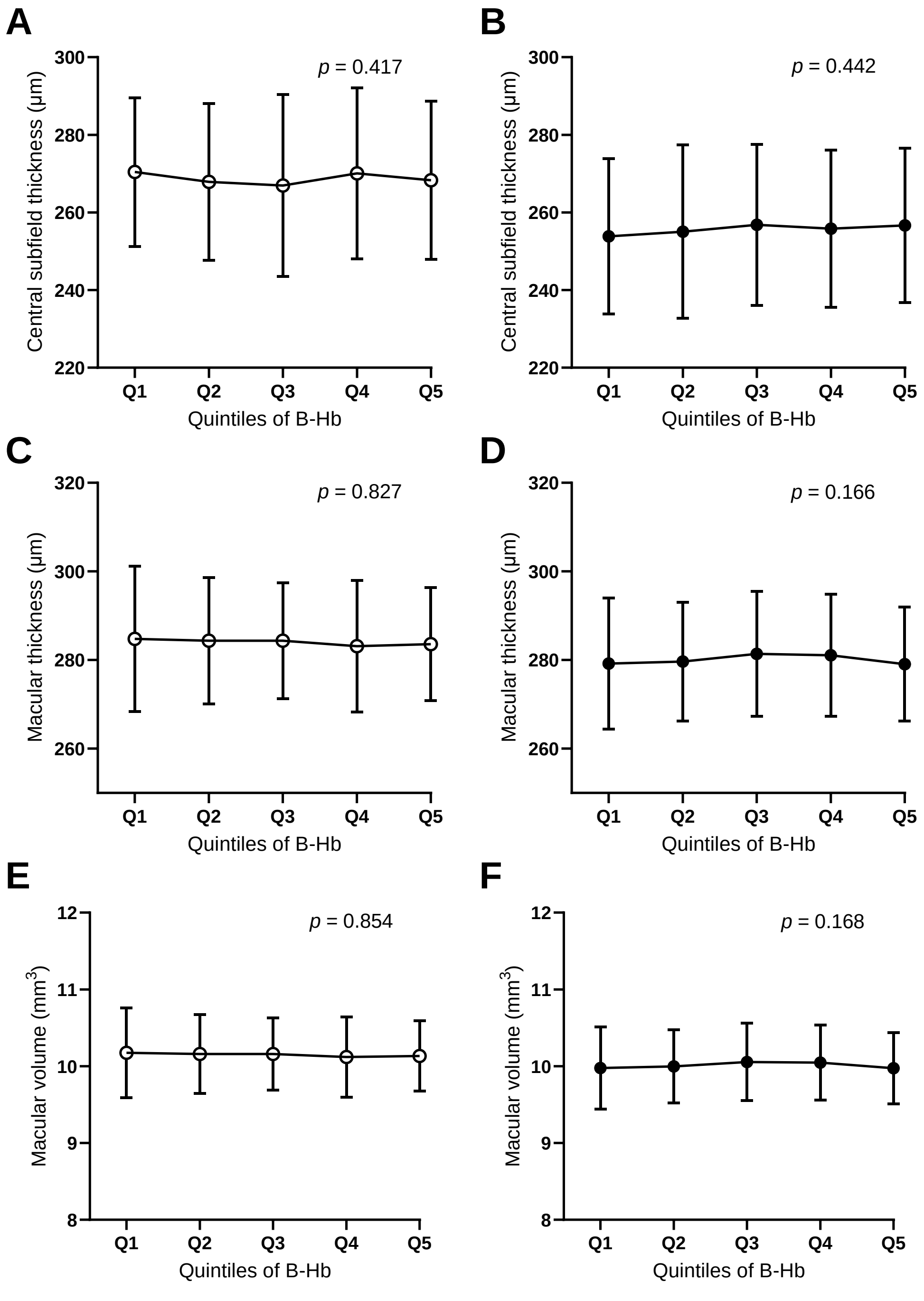

Figure S3. Association of macular parameters in sex-specific Hb quintiles. Central subfield thickness (**A**, **B**), Macular thickness (**C**, **D**) and Macular volume (**E**, **F**) in Hb quintiles for males (white dot; **A, C, E**) and females (black dot; **B, D, F**). The values are mean with (SD) or percentages. *P* is given for comparison over the Hb quintiles in one-way ANOVA. Q, quintile; B-Hb, blood hemoglobin.
